# μ-4,4′-Bipyridine-bis­[aqua­(4-hy­droxy­pyridine-2,6-dicarboxyl­ato)copper(II)]

**DOI:** 10.1107/S1600536812004758

**Published:** 2012-02-24

**Authors:** Xiao-Li Chen, Ya-Li Qiao, Lou-Jun Gao, Hua-Li Cui, Mei-Li Zhang

**Affiliations:** a Shaanxi Key Laboratory of Chemical Reaction Engineering, Department of Chemistry and Chemical Engineering, Yan’an University, Yan’an, Shaanxi 716000, People’s Republic of China

## Abstract

The title compound, [Cu_2_(C_7_H_3_NO_5_)_2_(C_10_H_8_N_2_)(H_2_O)_2_], exhibits a centrosymmetric binuclear molecule. Each completely deprotonated 4-hy­droxy­pyridine-2,6-dicarb­oxy­lic acid mol­ecule assumes a tridentate chelating coordination mode. The square-pyramidal coordination geometry around the Cu^II^ ion is completed by the bridging bipyridine ligand and an apical water molecule. Adjacent complexes are connected *via* O—H⋯O and C—H⋯O hydrogen bonds to generate a three-dimensional supra­molecular structure.

## Related literature

For related literature on the construction of supra­molecular structures, see: Robin & Fromm (2006 ▶); Desiraju (1989 ▶). For compounds using heterocyclic carb­oxy­lic acids such as pyridine-, pyrazole- and imidazole­carb­oxy­lic acids as building blocks, see: Lin *et al.* (1998 ▶); Zhao *et al.* (2003 ▶); Pan *et al.* (2000 ▶); Liu *et al.* (2004 ▶); Mahata & Natarajan (2005 ▶); Panagiotis *et al.* (2005 ▶).
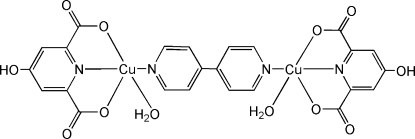


## Experimental

### 

#### Crystal data

[Cu_2_(C_7_H_3_NO_5_)_2_(C_10_H_8_N_2_)(H_2_O)_2_]
*M* *_r_* = 681.50
Monoclinic, 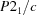

*a* = 8.3945 (9) Å
*b* = 18.433 (2) Å
*c* = 7.8686 (10) Å
β = 100.044 (2)°
*V* = 1198.9 (2) Å^3^
*Z* = 2
Mo *K*α radiation
μ = 1.85 mm^−1^
*T* = 296 K
0.30 × 0.25 × 0.25 mm

#### Data collection

Bruker SMART 1000 diffractometer
Absorption correction: multi-scan (*SADABS*; Sheldrick, 1996 ▶) *T*_min_ = 0.579, *T*_max_ = 0.629
6528 measured reflections
2433 independent reflections
1972 reflections with *I* > 2σ(*I*)
*R*_int_ = 0.041

#### Refinement

*R*[*F* ^2^ > 2σ(*F* ^2^)] = 0.045
*wR*(*F* ^2^) = 0.103
*S* = 1.09
2433 reflections
197 parameters
2 restraints
H atoms treated by a mixture of independent and constrained refinement
Δρ_max_ = 0.44 e Å^−3^
Δρ_min_ = −0.60 e Å^−3^

### 

Data collection: *SMART* (Bruker, 1997 ▶); cell refinement: *SAINT* (Bruker, 1997 ▶); data reduction: *SAINT*; program(s) used to solve structure: *SHELXS97* (Sheldrick, 2008 ▶); program(s) used to refine structure: *SHELXL97* (Sheldrick, 2008 ▶); molecular graphics: *SHELXTL* (Sheldrick, 2008 ▶); software used to prepare material for publication: *SHELXL97*.

## Supplementary Material

Crystal structure: contains datablock(s) I, global. DOI: 10.1107/S1600536812004758/ff2054sup1.cif

Structure factors: contains datablock(s) I. DOI: 10.1107/S1600536812004758/ff2054Isup2.hkl

Additional supplementary materials:  crystallographic information; 3D view; checkCIF report

## Figures and Tables

**Table 1:** Selected bond lengths (Å)

Cu1—N1	1.888 (3)
Cu1—N2	1.944 (3)
Cu1—O1	1.996 (2)
Cu1—O4	2.011 (2)
Cu1—O6	2.399 (3)

**Table 2:** Hydrogen-bond geometry (Å, °)

*D*—H⋯*A*	*D*—H	H⋯*A*	*D*⋯*A*	*D*—H⋯*A*
O3—H3⋯O6^i^	0.82	1.86	2.670 (3)	169
O6—H6*B*⋯O4^ii^	0.80 (2)	2.54 (3)	3.185 (3)	139 (3)
O6—H6*B*⋯O5^ii^	0.80 (2)	2.17 (2)	2.948 (3)	163 (4)
C12—H12⋯O3^iii^	0.93	2.58	3.246 (4)	129
C8—H8⋯O1	0.93	2.43	3.003 (4)	120
C12—H12⋯O4	0.93	2.59	3.142 (4)	118

Symmetry codes: (i) 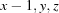; (ii) 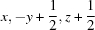; (iii) 
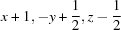
.

**Fig. 1.**
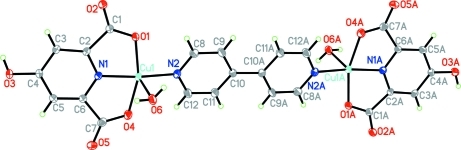
The molecular structure and labeling of the title compound. Displacement ellipsoids are drawn at the 30% probability level. [Symmetry code: (A) 2 - x, 1 - y, -z]

**Fig. 2.**
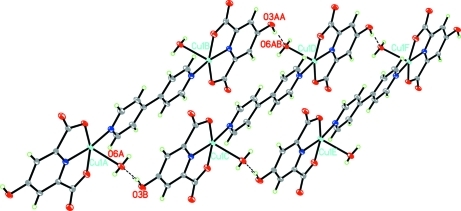
The one-dimensional supramolecular chain formed via hydrogen bonding interactions. Dashed lines denote hydrogen bonds.

**Fig. 3.**
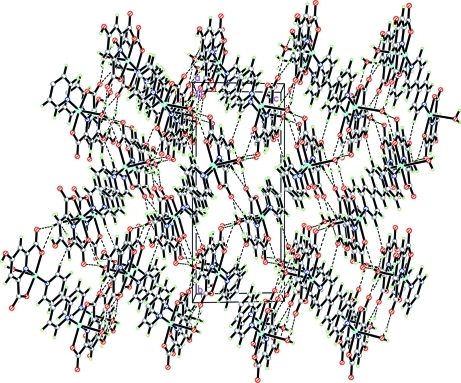
The three-dimensional supramolecular structure, viewed in the ac plane, linked via hydrogen bonding interactions. Dashed lines denote hydrogen bonds.

## supplementary crystallographic information

### Comment

The self-assembled construction of supramolecular structure is of current interest because controlling the molecular organization in the solid state can lead to materials with novel structure and promising properties (Desiraju, 1989; Robin & Fromm, 2006). Supramolecular chemistry uses molecular recognition processes that rely heavily on the understanding of the recognition properties of the functional groups involved in these interactions. Recently, increasing investigations have been focused on the constructions of supramolecular structure using heterocyclic carboxylic acids such as pyridine- (Lin *et al.*, 1998; Zhao *et al.*,2003), pyrazole- (Pan *et al.*, 2000), and imidazole-carboxylic acids (Liu *et al.*, 2004; Mahata & Natarajan, 2005; Panagiotis *et al.*, 2005) as building blocks. These building blocks contain multi-oxygen and N atoms and can coordinate with metal ions in different ways, resulting in the formations of various metal–organic frameworks with specific topologies and useful properties. In this aspect, 4-hydroxypyridine-2,6-dicarboxylic acid (cam), which has six potential donor atoms, is a quite versatile ligand for the construction of metal–organic hybrid compounds. Herein we hydrothermally synthesized the title compound, which exhibits a binuclear structure (Fig. 1). The asymmetric unit consists of a Cu^2+^ ion, one cam^2-^ ion, half 4,4'-bipy ligand, one coordinated water molecule. It is worth noting that each completely deprotonated cam^2-^ ion coordinates one Cu^2+^ ion in a tridentate chelating coordination mode (Scheme 1). Interestingly, the adjacent binuclear complexes form a one-dimensional supramolecular chain *via* O3—H3···O6 hydrogen bonding interaction (Fig. 2), which is further involved in a three-dimensional supramolecular structure connected *via* O—H···O and C—H···O hydrogen bonding interactions (Fig. 3).

### Experimental

The compound (I) was prepared by hydrothermal method. A mixture of CuSO_4_.5H_2_O (0.10 mmol), 4,4'-bipyridine (0.10 mmol), 4-hydroxypyridine-2,6-dicarboxylic acid (cam 0.10 mmol) and water (10 ml) was stirred for 30 min. The mixture was then transferred to a 23 ml Teflon-lined autoclave and kept at 433 K for 72 h under autogenous pressure. Then the mixture was cooled to room temperature slowly. Blue single crystals of the title compound suitable for X-ray analysis were obtained from the reaction mixture.

### Refinement

The H atoms of phenyl ring were included in the riding approximation with C—H = 0.93 Å, and with *U*_iso_(H) = 1.2*U*_eq_(C). The H atoms attached to O were located from a difference Fourier map and refined isotropically to O—H = 0.82 Å, with *U*_iso_(H) = 1.5*U*_eq_(O).

### Crystal data

**Table:**

[Cu_2_(C_7_H_3_NO_5_)_2_(C_10_H_8_N_2_)(H_2_O)_2_]	*F*(000) = 688
*M**_r_* = 681.50	*D*_x_ = 1.888 Mg m^−^^3^
Monoclinic, *P*2_1_/*c*	Mo *K*α radiation, λ = 0.71073 Å
Hall symbol: -P 2ybc	Cell parameters from 2433 reflections
*a* = 8.3945 (9) Å	θ = 2.2–26.4°
*b* = 18.433 (2) Å	µ = 1.85 mm^−^^1^
*c* = 7.8686 (10) Å	*T* = 296 K
β = 100.044 (2)°	Prism, blue
*V* = 1198.9 (2) Å^3^	0.30 × 0.25 × 0.25 mm
*Z* = 2	

### Data collection

**Table:**

Bruker SMART 1000 diffractometer	2433 independent reflections
Radiation source: fine-focus sealed tube	1972 reflections with *I* > 2σ(*I*)
Graphite monochromator	*R*_int_ = 0.041
φ and ω scans	θ_max_ = 26.4°, θ_min_ = 2.2°
Absorption correction: multi-scan (*SADABS*; Sheldrick, 1996)	*h* = −10→10
*T*_min_ = 0.579, *T*_max_ = 0.629	*k* = −20→23
6528 measured reflections	*l* = −8→9

### Refinement

**Table:**

Refinement on *F*^2^	Primary atom site location: structure-invariant direct methods
Least-squares matrix: full	Secondary atom site location: difference Fourier map
*R*[*F*^2^ > 2σ(*F*^2^)] = 0.045	Hydrogen site location: inferred from neighbouring sites
*wR*(*F*^2^) = 0.103	H atoms treated by a mixture of independent and constrained refinement
*S* = 1.09	*w* = 1/[σ^2^(*F*_o_^2^) + (0.0447*P*)^2^] where *P* = (*F*_o_^2^ + 2*F*_c_^2^)/3
2433 reflections	(Δ/σ)_max_ = 0.001
197 parameters	Δρ_max_ = 0.44 e Å^−^^3^
2 restraints	Δρ_min_ = −0.60 e Å^−^^3^

### Special details

**Table:**

Geometry. All e.s.d.'s (except the e.s.d. in the dihedral angle between two l.s. planes) are estimated using the full covariance matrix. The cell e.s.d.'s are taken into account individually in the estimation of e.s.d.'s in distances, angles and torsion angles; correlations between e.s.d.'s in cell parameters are only used when they are defined by crystal symmetry. An approximate (isotropic) treatment of cell e.s.d.'s is used for estimating e.s.d.'s involving l.s. planes.
Refinement. Refinement of *F*^2^ against ALL reflections. The weighted *R*-factor *wR* and goodness of fit *S* are based on *F*^2^, conventional *R*-factors *R* are based on *F*, with *F* set to zero for negative *F*^2^. The threshold expression of *F*^2^ > σ(*F*^2^) is used only for calculating *R*-factors(gt) *etc*. and is not relevant to the choice of reflections for refinement. *R*-factors based on *F*^2^ are statistically about twice as large as those based on *F*, and *R*-factors based on ALL data will be even larger.

### Fractional atomic coordinates and isotropic or equivalent isotropic displacement parameters (Å^2^)

**Table:**

	*x*	*y*	*z*	*U*_iso_*/*U*_eq_	
Cu1	0.47889 (5)	0.38222 (2)	0.23311 (6)	0.03017 (17)	
N1	0.2865 (3)	0.34582 (14)	0.2945 (4)	0.0261 (6)	
N2	0.6518 (3)	0.42601 (15)	0.1330 (4)	0.0300 (7)	
O1	0.3886 (3)	0.47502 (11)	0.3063 (3)	0.0332 (6)	
O2	0.1826 (3)	0.51815 (12)	0.4202 (4)	0.0377 (7)	
O3	−0.1274 (3)	0.26614 (12)	0.4182 (4)	0.0463 (8)	
H3	−0.1858	0.2984	0.4443	0.069*	
O4	0.4981 (3)	0.27793 (12)	0.1646 (4)	0.0386 (7)	
O5	0.3903 (3)	0.16954 (12)	0.1994 (4)	0.0428 (7)	
C1	0.2557 (4)	0.46880 (17)	0.3631 (5)	0.0280 (8)	
C2	0.1880 (4)	0.39256 (16)	0.3559 (4)	0.0245 (7)	
C3	0.0461 (4)	0.36900 (17)	0.4026 (5)	0.0292 (8)	
H3A	−0.0223	0.4010	0.4462	0.035*	
C4	0.0081 (4)	0.29506 (17)	0.3821 (5)	0.0305 (8)	
C5	0.1158 (4)	0.24840 (18)	0.3192 (5)	0.0318 (8)	
H5	0.0925	0.1992	0.3060	0.038*	
C6	0.2553 (4)	0.27552 (17)	0.2772 (5)	0.0266 (8)	
C7	0.3911 (4)	0.23570 (19)	0.2084 (5)	0.0316 (8)	
C8	0.6991 (4)	0.49448 (19)	0.1714 (5)	0.0369 (9)	
H8	0.6384	0.5229	0.2341	0.044*	
C9	0.8328 (4)	0.52411 (18)	0.1222 (5)	0.0363 (9)	
H9	0.8621	0.5715	0.1542	0.044*	
C10	0.9259 (4)	0.48484 (17)	0.0252 (4)	0.0276 (8)	
C11	0.8717 (4)	0.41528 (19)	−0.0187 (5)	0.0377 (10)	
H11	0.9275	0.3867	−0.0861	0.045*	
C12	0.7374 (5)	0.38763 (18)	0.0353 (5)	0.0399 (10)	
H12	0.7044	0.3407	0.0030	0.048*	
O6	0.6528 (3)	0.35779 (13)	0.5027 (4)	0.0359 (6)	
H6A	0.689 (4)	0.3975 (13)	0.543 (5)	0.043*	
H6B	0.593 (4)	0.3429 (19)	0.564 (4)	0.043*	

### Atomic displacement parameters (Å^2^)

**Table:**

	*U*^11^	*U*^22^	*U*^33^	*U*^12^	*U*^13^	*U*^23^
Cu1	0.0234 (3)	0.0274 (3)	0.0441 (3)	−0.00220 (18)	0.0182 (2)	−0.00282 (19)
N1	0.0228 (15)	0.0241 (14)	0.0344 (17)	0.0011 (12)	0.0136 (13)	−0.0017 (13)
N2	0.0243 (15)	0.0311 (16)	0.0384 (18)	0.0006 (12)	0.0161 (14)	0.0009 (13)
O1	0.0278 (13)	0.0223 (12)	0.0547 (17)	−0.0030 (10)	0.0215 (12)	−0.0014 (11)
O2	0.0336 (14)	0.0254 (13)	0.0598 (19)	−0.0023 (11)	0.0238 (13)	−0.0090 (12)
O3	0.0297 (14)	0.0281 (13)	0.088 (2)	−0.0026 (11)	0.0305 (15)	0.0064 (15)
O4	0.0310 (14)	0.0336 (14)	0.0572 (18)	−0.0015 (11)	0.0239 (13)	−0.0100 (13)
O5	0.0412 (16)	0.0261 (14)	0.0638 (19)	0.0039 (12)	0.0165 (14)	−0.0095 (13)
C1	0.0260 (18)	0.0241 (17)	0.035 (2)	−0.0014 (14)	0.0094 (16)	−0.0017 (15)
C2	0.0203 (17)	0.0250 (17)	0.0291 (19)	0.0006 (13)	0.0071 (14)	−0.0002 (14)
C3	0.0216 (18)	0.0296 (18)	0.038 (2)	0.0019 (14)	0.0108 (16)	0.0018 (16)
C4	0.0226 (18)	0.0265 (18)	0.044 (2)	−0.0029 (14)	0.0109 (16)	0.0042 (16)
C5	0.0311 (19)	0.0220 (17)	0.045 (2)	−0.0010 (15)	0.0144 (17)	0.0022 (16)
C6	0.0243 (18)	0.0223 (17)	0.035 (2)	0.0001 (14)	0.0091 (15)	−0.0002 (15)
C7	0.029 (2)	0.034 (2)	0.033 (2)	0.0039 (16)	0.0077 (16)	−0.0084 (16)
C8	0.037 (2)	0.0298 (19)	0.050 (3)	0.0017 (16)	0.0242 (19)	−0.0058 (18)
C9	0.037 (2)	0.0253 (18)	0.051 (2)	−0.0088 (16)	0.0220 (19)	−0.0089 (17)
C10	0.0269 (19)	0.0270 (18)	0.032 (2)	−0.0004 (15)	0.0120 (16)	0.0057 (15)
C11	0.034 (2)	0.0312 (19)	0.055 (3)	−0.0020 (16)	0.0280 (19)	−0.0057 (18)
C12	0.040 (2)	0.0288 (19)	0.057 (3)	−0.0072 (17)	0.027 (2)	−0.0071 (18)
O6	0.0350 (16)	0.0296 (13)	0.0482 (18)	−0.0092 (11)	0.0211 (13)	−0.0041 (12)

### Geometric parameters (Å, º)

**Table:**

Cu1—N1	1.888 (3)	C3—C4	1.403 (4)
Cu1—N2	1.944 (3)	C3—H3A	0.9300
Cu1—O1	1.996 (2)	C4—C5	1.400 (4)
Cu1—O4	2.011 (2)	C5—C6	1.365 (4)
Cu1—O6	2.399 (3)	C5—H5	0.9300
N1—C6	1.324 (4)	C6—C7	1.532 (4)
N1—C2	1.341 (4)	C8—C9	1.363 (4)
N2—C12	1.342 (4)	C8—H8	0.9300
N2—C8	1.342 (4)	C9—C10	1.388 (4)
O1—C1	1.277 (4)	C9—H9	0.9300
O2—C1	1.226 (4)	C10—C11	1.384 (5)
O3—C4	1.330 (4)	C10—C10^i^	1.480 (6)
O3—H3	0.8200	C11—C12	1.370 (5)
O4—C7	1.280 (4)	C11—H11	0.9300
O5—C7	1.222 (4)	C12—H12	0.9300
C1—C2	1.513 (4)	O6—H6A	0.834 (18)
C2—C3	1.377 (4)	O6—H6B	0.804 (18)
			
N1—Cu1—N2	169.74 (13)	O3—C4—C3	123.4 (3)
N1—Cu1—O1	81.12 (10)	C5—C4—C3	119.3 (3)
N2—Cu1—O1	96.27 (10)	C6—C5—C4	119.7 (3)
N1—Cu1—O4	80.85 (10)	C6—C5—H5	120.2
N2—Cu1—O4	100.83 (10)	C4—C5—H5	120.2
O1—Cu1—O4	161.60 (9)	N1—C6—C5	119.7 (3)
N1—Cu1—O6	97.05 (10)	N1—C6—C7	111.0 (3)
N2—Cu1—O6	93.09 (10)	C5—C6—C7	129.2 (3)
O1—Cu1—O6	96.24 (10)	O5—C7—O4	126.0 (3)
O4—Cu1—O6	89.58 (10)	O5—C7—C6	120.1 (3)
C6—N1—C2	122.8 (3)	O4—C7—C6	113.8 (3)
C6—N1—Cu1	119.0 (2)	N2—C8—C9	122.6 (3)
C2—N1—Cu1	118.2 (2)	N2—C8—H8	118.7
C12—N2—C8	117.3 (3)	C9—C8—H8	118.7
C12—N2—Cu1	121.7 (2)	C8—C9—C10	121.2 (3)
C8—N2—Cu1	120.8 (2)	C8—C9—H9	119.4
C1—O1—Cu1	115.01 (19)	C10—C9—H9	119.4
C4—O3—H3	109.5	C11—C10—C9	115.4 (3)
C7—O4—Cu1	114.6 (2)	C11—C10—C10^i^	122.6 (4)
O2—C1—O1	125.8 (3)	C9—C10—C10^i^	122.1 (4)
O2—C1—C2	119.6 (3)	C12—C11—C10	121.3 (3)
O1—C1—C2	114.6 (3)	C12—C11—H11	119.3
N1—C2—C3	120.7 (3)	C10—C11—H11	119.3
N1—C2—C1	111.0 (3)	N2—C12—C11	122.2 (3)
C3—C2—C1	128.3 (3)	N2—C12—H12	118.9
C2—C3—C4	117.8 (3)	C11—C12—H12	118.9
C2—C3—H3A	121.1	Cu1—O6—H6A	107 (3)
C4—C3—H3A	121.1	Cu1—O6—H6B	104 (3)
O3—C4—C5	117.3 (3)	H6A—O6—H6B	107 (4)
			
N2—Cu1—N1—C6	103.6 (6)	O1—C1—C2—N1	1.8 (5)
O1—Cu1—N1—C6	179.6 (3)	O2—C1—C2—C3	1.9 (6)
O4—Cu1—N1—C6	3.3 (3)	O1—C1—C2—C3	−178.0 (3)
O6—Cu1—N1—C6	−85.2 (3)	N1—C2—C3—C4	−0.5 (5)
N2—Cu1—N1—C2	−77.2 (7)	C1—C2—C3—C4	179.2 (3)
O1—Cu1—N1—C2	−1.2 (3)	C2—C3—C4—O3	−178.2 (3)
O4—Cu1—N1—C2	−177.5 (3)	C2—C3—C4—C5	1.2 (5)
O6—Cu1—N1—C2	94.1 (3)	O3—C4—C5—C6	178.9 (3)
N1—Cu1—N2—C12	−93.6 (7)	C3—C4—C5—C6	−0.6 (6)
O1—Cu1—N2—C12	−168.3 (3)	C2—N1—C6—C5	1.6 (5)
O4—Cu1—N2—C12	4.9 (3)	Cu1—N1—C6—C5	−179.3 (3)
O6—Cu1—N2—C12	95.1 (3)	C2—N1—C6—C7	−178.6 (3)
N1—Cu1—N2—C8	92.1 (7)	Cu1—N1—C6—C7	0.6 (4)
O1—Cu1—N2—C8	17.4 (3)	C4—C5—C6—N1	−0.8 (5)
O4—Cu1—N2—C8	−169.4 (3)	C4—C5—C6—C7	179.4 (4)
O6—Cu1—N2—C8	−79.2 (3)	Cu1—O4—C7—O5	−170.6 (3)
N1—Cu1—O1—C1	2.3 (3)	Cu1—O4—C7—C6	9.4 (4)
N2—Cu1—O1—C1	172.2 (3)	N1—C6—C7—O5	173.3 (3)
O4—Cu1—O1—C1	13.9 (5)	C5—C6—C7—O5	−6.9 (6)
O6—Cu1—O1—C1	−93.9 (3)	N1—C6—C7—O4	−6.8 (5)
N1—Cu1—O4—C7	−7.3 (3)	C5—C6—C7—O4	173.1 (4)
N2—Cu1—O4—C7	−177.1 (3)	C12—N2—C8—C9	−3.2 (6)
O1—Cu1—O4—C7	−19.0 (5)	Cu1—N2—C8—C9	171.4 (3)
O6—Cu1—O4—C7	89.9 (3)	N2—C8—C9—C10	1.5 (6)
Cu1—O1—C1—O2	177.3 (3)	C8—C9—C10—C11	1.0 (6)
Cu1—O1—C1—C2	−2.8 (4)	C8—C9—C10—C10^i^	−178.2 (4)
C6—N1—C2—C3	−0.9 (5)	C9—C10—C11—C12	−1.6 (6)
Cu1—N1—C2—C3	179.9 (3)	C10^i^—C10—C11—C12	177.5 (4)
C6—N1—C2—C1	179.3 (3)	C8—N2—C12—C11	2.5 (6)
Cu1—N1—C2—C1	0.1 (4)	Cu1—N2—C12—C11	−172.0 (3)
O2—C1—C2—N1	−178.2 (3)	C10—C11—C12—N2	−0.1 (6)

Symmetry code: (i) −*x*+2, −*y*+1, −*z*.

### Hydrogen-bond geometry (Å, º)

**Table:**

*D*—H···*A*	*D*—H	H···*A*	*D*···*A*	*D*—H···*A*
O3—H3···O6^ii^	0.82	1.86	2.670 (3)	169
O6—H6*B*···O4^iii^	0.80 (2)	2.54 (3)	3.185 (3)	139 (3)
O6—H6*B*···O5^iii^	0.80 (2)	2.17 (2)	2.948 (3)	163 (4)
C12—H12···O3^iv^	0.93	2.58	3.246 (4)	129
C8—H8···O1	0.93	2.43	3.003 (4)	120
C12—H12···O4	0.93	2.59	3.142 (4)	118

Symmetry codes: (ii) *x*−1, *y*, *z*; (iii) *x*, −*y*+1/2, *z*+1/2; (iv) *x*+1, −*y*+1/2, *z*−1/2.
